# Artificial intelligence in healthcare education: evaluating the accuracy of ChatGPT, Copilot, and Google Gemini in cardiovascular pharmacology

**DOI:** 10.3389/fmed.2025.1495378

**Published:** 2025-02-19

**Authors:** Ibrahim M. Salman, Omar Z. Ameer, Mohammad A. Khanfar, Yee-Hsee Hsieh

**Affiliations:** ^1^Department of Pharmaceutical Sciences, College of Pharmacy, Alfaisal University, Riyadh, Saudi Arabia; ^2^Division of Pulmonary, Critical Care, and Sleep Medicine, School of Medicine, Case Western Reserve University, Cleveland, OH, United States

**Keywords:** ChatGPT, Copilot, Google Gemini, cardiovascular pharmacology, pharmacy and medicine, medical education

## Abstract

**Background:**

Artificial intelligence (AI) is revolutionizing medical education; however, its limitations remain underexplored. This study evaluated the accuracy of three generative AI tools—ChatGPT-4, Copilot, and Google Gemini—in answering multiple-choice questions (MCQ) and short-answer questions (SAQ) related to cardiovascular pharmacology, a key subject in healthcare education.

**Methods:**

Using free versions of each AI tool, we administered 45 MCQs and 30 SAQs across three difficulty levels: easy, intermediate, and advanced. AI-generated answers were reviewed by three pharmacology experts. The accuracy of MCQ responses was recorded as correct or incorrect, while SAQ responses were rated on a 1–5 scale based on relevance, completeness, and correctness.

**Results:**

ChatGPT, Copilot, and Gemini demonstrated high accuracy scores in easy and intermediate MCQs (87–100%). While all AI models showed a decline in performance on the advanced MCQ section, only Copilot (53% accuracy) and Gemini (20% accuracy) had significantly lower scores compared to their performance on easy-intermediate levels. SAQ evaluations revealed high accuracy scores for ChatGPT (overall 4.7 ± 0.3) and Copilot (overall 4.5 ± 0.4) across all difficulty levels, with no significant differences between the two tools. In contrast, Gemini’s SAQ performance was markedly lower across all levels (overall 3.3 ± 1.0).

**Conclusion:**

ChatGPT-4 demonstrates the highest accuracy in addressing both MCQ and SAQ cardiovascular pharmacology questions, regardless of difficulty level. Copilot ranks second after ChatGPT, while Google Gemini shows significant limitations in handling complex MCQs and providing accurate responses to SAQ-type questions in this field. These findings can guide the ongoing refinement of AI tools for specialized medical education.

## 1 Introduction

The emergence of generative artificial intelligence (AI) models, including ChatGPT (Chat Generative Pre-Trained Transformer), Microsoft Copilot, and, more recently, Google Gemini, has significantly influenced the educational landscape across various disciplines, particularly in healthcare and healthcare education. These AI platforms are increasingly utilized by students in medical, pharmacy, nursing, dental and allied health programs to assist with studying, personal tutoring, exam preparation, and completing assignments, due to their remarkable ability to provide real-time assistance and tailored information relevant to coursework and research activities (1, 2). Recent advancements in natural language processing (NLP) have enabled these tools to generate more contextually accurate and detailed responses to a wide range of queries (3, 4). As large language models (LLMs), these tools are trained on vast datasets, which enables them to process questions and provide relevant, up-to-date answers (3–5).

ChatGPT, developed by OpenAI (6), Copilot (previously known as Bing Chat), a product of GitHub and Microsoft (7), and Google Gemini (previously known as Bard), created by Google DeepMind (8), represent three distinct approaches to generative AI. ChatGPT is a LLM that employs fine-tuning and Reinforcement Learning from Human Feedback (RLHF) and generates text based on prompts, relying on a vast dataset to provide coherent and contextually relevant responses (9). Copilot, which utilizes an evolving GPT model like ChatGPT, is designed as an AI-powered code completion tool that leverages machine learning to assist developers by predicting and suggesting code snippets (10). Google Gemini, known for integrating deep learning with LLMs, aims to provide accurate and insightful text responses in a variety of specialized fields (4, 11) and serve as a writing, planning, and learning assistant (12). Despite their different primary functions, all three tools have been adapted for educational purposes, including answering questions in specialized fields. Nonetheless, it remains unclear how effectively they can deliver concise and accurate scientific information. As AI continues to grow in influence, particularly in healthcare education, it is critical to systematically assess its accuracy and reliability in providing correct scientific information. Accordingly, active research in this area has the potential to optimize this emerging technology.

Cardiovascular pharmacology, a core subject in healthcare education, represents an ideal context for conducting such an evaluation. This subject involves understanding drug mechanisms, therapeutic uses, and the management of cardiovascular conditions. Mastery of both factual knowledge and the ability to apply that knowledge in clinical settings is essential for ensuring safe and effective healthcare practices. Given the complexity of this field, ensuring that students receive accurate and comprehensive information when using AI platforms is crucial for their development as future healthcare professionals (13). Research studies suggest that while these platforms can address straightforward factual queries (14, 15), their performance in handling more complex, subject-specific material, particularly in highly technical (16, 17) or medical domains (18–20), including cardiovascular medicine (21, 22), remains largely underexplored. Understanding the accuracy and limitations of these tools is essential for educators to integrate them effectively into curricula, ensuring students receive reliable, high-quality support. Accordingly, our study aimed to systematically compare the accuracy and correctness of answers provided by ChatGPT, Copilot, and Google Gemini in response to multiple-choice questions (MCQs) and short-answer questions (SAQs) of varying difficulty levels in the field of cardiovascular pharmacology to gain insights into the effectiveness of these tools as educational resources for healthcare students.

## 2 Methods

### 2.1 Study sample

**Figure 1:**
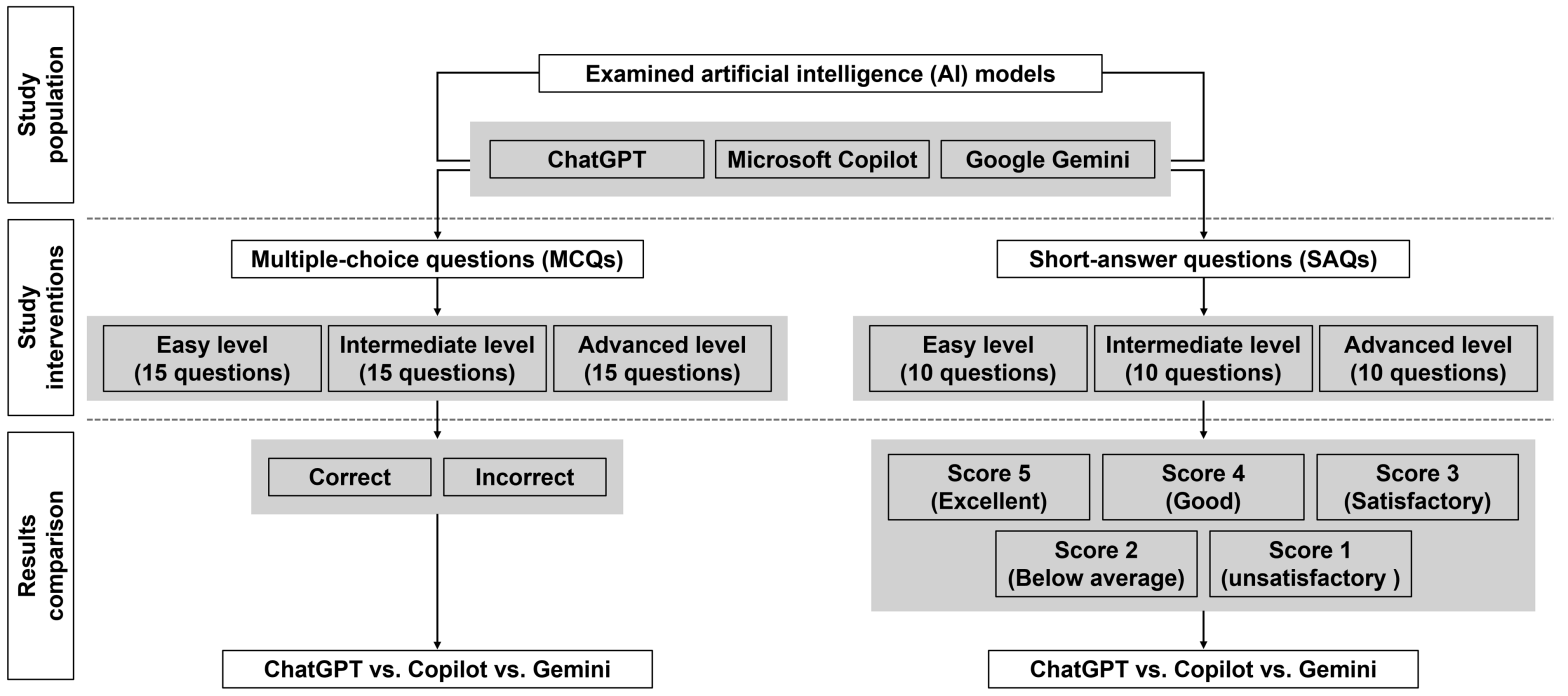
Summary flowchart of the study.

An experimental study was conducted using the free-access versions of three AI-powered tools: ChatGPT (GPT-4o mini, OpenAI), Microsoft Copilot (GPT-4 Turbo, GitHub Copilot), and Google Gemini (Gemini 1.5, Google DeepMind). These tools were all accessed in September 2024 and tasked with answering MCQs and SAQs across three different difficulty levels: easy, intermediate, and advanced (Figure 1). No ethical approval was sought, as this study did not involve any human participants.

### 2.2 Study design

Test questions were designed to assess various domains, including basic recall and understanding of cardiovascular pharmacology concepts, application and integration of pharmacology knowledge, and analysis, synthesis, and evaluation of complex pharmacological scenarios. Easy questions required simple recall of facts, such as definitions and basic applications. Intermediate questions focused on deeper understanding, explanations, and comparisons. Advanced questions demanded critical thinking, knowledge integration, and analysis of complex scenarios, as well as explanations of advanced concepts.

All test questions and their model answers were prepared by an experienced pharmacology professor, the first author (I.S.) of this paper. Two additional pharmacology professors, the second (O.A.) and third (M.K.) authors, validated the questions to ensure clarity and appropriate difficulty levels. I.S., O.A., and M.K. are all licensed pharmacists with extensive experience in pharmacology education at the university level. Moreover, I.S. and O.A. specialize in cardiovascular and autonomic pharmacology and physiology, which ensured a rigorous evaluation of the AI-generated responses. All questions used in this study were original and did not utilize any online practice questions or question banks.

To maintain the integrity of the AI tools’ performance and minimize systemic errors, only one prompt was used per test, and no additional prompts were introduced. A pilot study was conducted to ensure the prompts were understood and correctly used to input questions into the AI tools (see Supplementary material).

Each AI tool was subjected to two tests (Figure 1). The same questions were manually input into the AI chat box three times—once for each tool—and the provided answers were recorded. The first test consisted of 45 MCQs, with 15 questions per difficulty level and 5 possible answer choices (with only one correct answer). Easy and intermediate MCQs required straightforward selection of the correct answer, while advanced MCQs posed more complex scenarios to select the best answer (see Supplementary material for examples). The second test involved 30 short-answer, open-ended questions, with 10 questions per difficulty level (see Supplementary material for examples). To ensure a comprehensive and unbiased evaluation, all AI-generated SAQ answers were anonymized and independently rated by the three pharmacology professors mentioned above.

### 2.3 Data processing and analysis

Answers provided by all AI models were reviewed by I.S., O.A., and M.K. and compared against the model answers. For MCQ-type questions, responses from each AI tool were evaluated as correct or incorrect, and percentage scores were reported for each difficulty level and overall. For SAQ-type questions, answers were graded on a 1–5 scale based on accuracy, relevance, and completeness (see below). Both raw and percentage scores were reported.

- Score 5 (Excellent): Demonstrates comprehensive understanding, fully addresses all parts of the question, shows strong critical thinking, and contains no errors.
- Score 4 (Good): Shows solid understanding, addresses most of the question with minor errors, and includes some critical insight.
- Score 3 (Satisfactory): Indicates reasonable understanding, addresses key points but lacks depth, with some errors.
- Score 2 (Below average): Contains gaps in understanding, is incomplete or superficial, with notable errors.
- Score 1 (Unsatisfactory): Displays little to no understanding, mostly irrelevant information, and numerous errors.

All data were analyzed using GraphPad Prism (v9, GraphPad Software Inc., La Jolla, CA, USA). For categorical data derived from responses to MCQ-type questions, a Chi-square test of independence was performed to assess the association between the type of AI model and question difficulty level. Following a significant result, *post hoc* pairwise comparisons were conducted to identify specific group differences. To account for multiple comparisons and control for Type I errors, the Bonferroni correction (23) was applied to adjust the significance levels. The corrected *p*-values were used to determine the significance of each pairwise comparison.

For SAQ scores, the rounded scores per difficulty level for each AI tool were compared between evaluators, and a Fleiss’ Kappa test (24) was applied to assess inter-rater reliability among the three evaluators, with values interpreted as follows: Kappa<0 indicates poor agreement, 0–0.20 slight agreement, 0.21–0.40 fair agreement, 0.41–0.60 moderate agreement, 0.61–0.80 substantial agreement, and 0.81–1.00 near perfect to perfect agreement. Scores provided by all raters were averaged prior to further analysis. The accuracy scores of responses from the three AI tools were compared across all three difficulty levels using a two-way analysis of variance (ANOVA) to assess the main effects of AI tool type and difficulty level, as well as their interaction. The overall scores of all three AI tools were compared using one-way ANOVA. *Post hoc* comparisons were performed using the Bonferroni correction (23) to adjust for multiple comparisons. A significance level of *p* ≤ 0.05 was used for all analyses.

## 3 Results

### 3.1 Accuracy of AI responses to MCQ tests

The numbers and percentages of correct and incorrect responses generated by each AI platform at each difficulty level, as well as overall, are provided in Supplementary Table S1.

Except for one advanced MCQ question, where Gemini did not provide an answer, all AI tools answered every question, with no blank responses recorded. The missing response from Gemini was marked as incorrect during evaluation.

**Figure 2:**
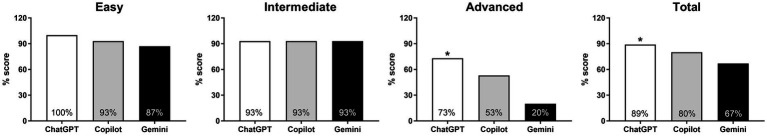
Pairwise comparison of AI models’ responses to MCQ-type questions, both overall and by difficulty level. *p < 0.05 for ChatGPT vs. Gemini.

The accuracy percentage scores for ChatGPT, Copilot, and Gemini were relatively similar (*p* > 0.999) on the easy and intermediate-level MCQ tests (Figure 2). For the easy MCQ test, ChatGPT scored 100%, followed by Copilot with 93% and Gemini with 87%, both falling within a narrow range of the highest possible scores (Figure 2). At the intermediate difficulty level, all three AI models achieved identical accuracy scores, with a staggering 93% score (Figure 2).

In the advanced MCQ test, however, all three models showed lower accuracy compared to previous levels. ChatGPT scored 73%, Copilot 53%, and Gemini 20% (Figure 2). Pairwise comparisons indicated that ChatGPT and Copilot had a relatively similar performance (*p* = 0.256), while ChatGPT’s performance was significantly higher than Gemini’s (*p* = 0.003), and Copilot also nearly outperformed Gemini (*p* = 0.058).

Overall, ChatGPT and Copilot’s performances were comparatively similar (*p* = 0.224), with Gemini demonstrating the lowest accuracy, particularly when compared to ChatGPT (*p* = 0.011) (Figure 2).

**Figure 3:**
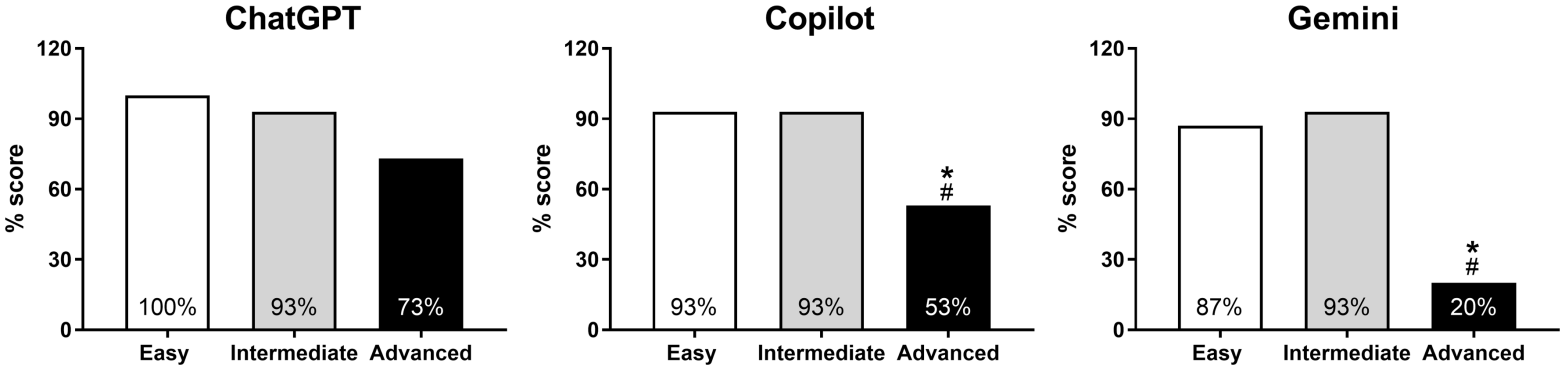
Pairwise comparison of responses to MCQ-type questions within each AI model based on difficulty levels. *p < 0.05 for easy vs. advanced and ^#^*p* < 0.05 for intermediate vs. advanced.

Within ChatGPT, the percentage scores for the MCQ questions at the easy and intermediate difficulty levels (93–100%) were numerically higher compared to those at the advanced level (73%), with the Chi-square *p*-value almost approaching statistical difference (*p* = 0.054) between these groups (Figure 3). In contrast, the high scores (87–93%) achieved by both Copilot and Gemini on the easy and intermediate-level questions were not replicated in the advanced section, with pairwise comparisons revealing significantly (all *p* < 0.05) lower scores (Copilot 53%, and Gemini 20%) in this section relative to both the easy and intermediate levels (Figure 3).

### 3.2 Accuracy of AI responses to SAQ tests

The raw accuracy scores and percentages for SAQ responses from each AI model at each difficulty level, as well as overall, are presented in Supplementary Table S2. Fleiss’ Kappa for inter-rater reliability was 0.873, indicating almost perfect agreement among all evaluators (*p* < 0.001). All SAQ-type questions were answered, and no AI tool failed to provide a response to any of the questions asked.

**Figure 4:**
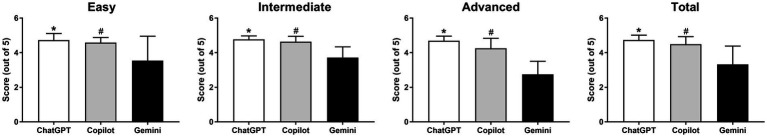
Pairwise comparison of AI models’ responses to SAQ-type questions, both overall and by difficulty level. *p < 0.05 for ChatGPT vs. Gemini and ^#^*p* < 0.05 for Copilot vs. Gemini.

The accuracy scores for ChatGPT and Copilot, across all levels of difficulty (minimum *p* = 0.397), as well as overall (*p* = 0.518), did not differ significantly (Figure 4). In contrast, Gemini consistently performed lower within each SAQ difficulty level (all *p* < 0.01) and overall (all *p* < 0.001) compared with both ChatGPT and Copilot (Figure 4).

**Figure 5:**
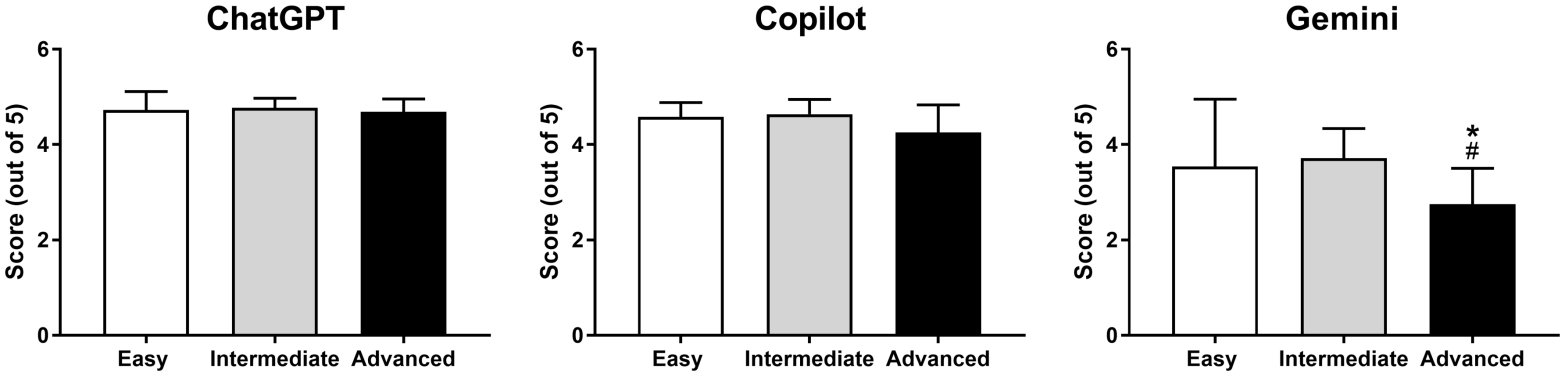
Pairwise comparison of responses to SAQ-type questions within each AI model based on difficulty levels. *p < 0.05 for easy vs. advanced and ^#^*p* < 0.05 for intermediate vs. advanced.

The data indicated that both ChatGPT and Copilot achieved consistently high accuracy scores across all SAQ difficulty levels, with no significant differences observed between different difficulty levels (minimum *p* = 0.545). In contrast, Gemini’s performance was markedly lower, particularly in the advanced SAQ section, where its accuracy scores were significantly lower (maximum *p* = 0.012) compared to both the easy and intermediate sections of the test (Figure 5). No significant interaction effect (*p* = 0.239) was observed between the independent variables (AI tool type and difficulty of the question).

## 4 Discussion

Understanding the strengths and limitations of AI-powered tools like ChatGPT, Microsoft Copilot, and Google Gemini is crucial as these platforms become more integrated into healthcare education. The findings of this study provide significant insights into the performance of these widely used generative AI models when applied in a healthcare education context, specifically within cardiovascular pharmacology. Given the increasing reliance on AI for student learning, these results have direct implications for how educators can integrate such tools into curricula. The main findings were as follows: (1) ChatGPT performed excellently in easy-intermediate level MCQs and in SAQs of all difficulty levels. However, it slightly, albeit insignificantly, underperformed in advanced MCQs; (2) Copilot similarly showed excellent performance in easy-intermediate level MCQs and in SAQs of all levels of difficulty, but it significantly underperformed in advanced MCQs; and (3) Google Gemini performed reasonably well in easy-intermediate level MCQs. However, its performance in advanced MCQs and SAQs of all difficulty levels was quite poor and fell far behind ChatGPT and Copilot. While AI platforms have demonstrated promising potential for delivering real-time assistance in educational environments, our results indicate some unignorable variability in their effectiveness, particularly as the complexity of the subject matter increases. Ongoing assessment and refinement of AI models to optimize their use as educational resources, especially in the highly technical field of healthcare, is key to enhancing their reliability across different disciplines.

Performance on MCQs revealed that ChatGPT, Copilot, and Gemini performed almost similarly when answering easy and intermediate-level MCQs. Both ChatGPT and Copilot demonstrated the highest accuracy in these sections. Google Gemini lagged slightly but remained within a similar performance range. These results suggest that for foundational and moderately complex factual knowledge, AI models can provide students with highly accurate responses. For instructors, these tools could be reliable for reinforcing basic pharmacology principles. Our data confirm previous studies that highlighted AI’s strength in handling basic factual queries (14, 15). However, the advanced MCQs revealed substantial performance gaps. ChatGPT maintained a relatively high accuracy score, but both Copilot and Gemini showed limitations, with Gemini performing notably worse. Collectively, these findings support the notion that while AI models excel at handling simpler tasks, their ability to navigate more complex questions and scenarios diminishes significantly as the difficulty increases, raising concerns about their use for advanced learning without proper human oversight. This may be attributed to the nature of advanced questions in cardiovascular pharmacology, which often require critical thinking, deeper knowledge integration, and the application of principles in clinical settings. Unlike basic recall questions, these advanced queries demand an in-depth understanding that current AI models may not yet be fully equipped to handle effectively. For students, this means that reliance on AI tools for advanced topics could lead to gaps in understanding, and instructors must ensure that these platforms are used in conjunction with traditional learning methods.

Interestingly, when all MCQ data were pooled, Copilot emerged as a competitive performer compared to ChatGPT, while Gemini earned the title of poorest performer. Roos and colleagues reported a similar level of accuracy between ChatGPT and Copilot in answering MCQs from the German Medical State Examinations (25), whereas Rossettini and colleagues demonstrated that Gemini had the lowest performance compared to ChatGPT and Copilot in answering MCQs routinely administered in the Italian standardized entrance examination for healthcare science degrees (26). It is therefore possible that the significant disparity between ChatGPT and Gemini on advanced-level MCQ-type questions points to differences in the underlying architecture and training of these AI models. While both are designed to process large datasets and generate coherent responses, ChatGPT’s language model, which is fine-tuned with human feedback (9), appears better suited to addressing complex medical scenarios. In contrast, Gemini, despite its advanced deep-learning capabilities (11), may not yet be optimized for domain-specific challenges like advanced pharmacology, limiting its effectiveness when used in this educational context.

The performance trends observed in the SAQs were partially consistent with the results from the MCQs. Unlike their performance on MCQs, both ChatGPT and Copilot showed remarkable performance across all SAQ difficulty levels, while Gemini consistently performed lower across all sections of the test. The almost perfect inter-rater reliability Fleiss’ Kappa score indicated that the evaluators agreed quite strongly on the accuracy and quality of the answers provided by the AI models, which further validated the observed performance differences. One of the most intriguing observations from the SAQ data was the lack of significant variation in performance across different difficulty levels for ChatGPT and Copilot. This suggests that these tools have a robust capability to respond accurately to the type of questions that require more detailed explanation, at least in the field of cardiovascular pharmacology, and that their high performance on SAQs may stem from their training on large and diverse datasets, which enables them to generate more contextually appropriate responses. For educators, this implies that ChatGPT and Copilot can be useful for question-based learning when detailed responses are needed.

It is noteworthy to mention that analyzing complex scenarios using the previous version of ChatGPT (ChatGPT-3.5) appeared to be less accurate and more challenging compared to Copilot and Gemini (10, 20, 27–29). However, clear improvements were noted with the latest ChatGPT-4 version (28, 30), which may explain the most impressive performance for ChatGPT in our study. Indeed, recent studies have shown relatively similar competence between ChatGPT and Copilot in answering open-ended questions in the medical field (31, 32), aligning with the findings of our present study, where both models demonstrated strong performance in answering SAQs across all difficulty levels. Nevertheless, it must be acknowledged that some studies have also reported instances where ChatGPT-4’s accuracy exceeded that of Copilot in specific scenarios, particularly in handling complex medical queries (15, 33). This suggests that while Copilot is competitive overall, there may be certain complex tasks or advanced cognitive faculties such as critical reasoning and clinical decision-making where ChatGPT-4 excels more noticeably. These specific areas were not directly tested in the current study but highlight the evolving capabilities of these AI tools as they continue to be refined. In contrast, Gemini’s marked underperformance in this section, particularly on the advanced SAQs, highlights significant limitations in this model’s ability to address complex, open-ended queries of this nature. The gap between Gemini and the other two tools could be explained by the distinct objectives of each AI model as alluded to earlier. Intriguingly, a similar lag in Google Gemini performance behind that of ChatGPT and/or Copilot in generating clinically based responses has been frequently reported (31–34). While ChatGPT and Copilot have been more widely adapted for general and educational purposes, Gemini is a newer tool that may be more specialized in other areas, which could explain its lower effectiveness in broad applications like medical education. Although refining Gemini’s training algorithms, particularly in handling medical topics that require not only factual knowledge but also the synthesis of information in real-world scenarios, may improve its performance, this was beyond the scope of the present study. The relatively similar overall performance between ChatGPT and Copilot, on the other hand, can be attributed to their shared use of the GPT-4 architecture, employing deep learning techniques to generate human-like text. However, while Copilot is tailored more for code generation, ChatGPT is optimized for a broader range of human-like text generation (10). Given the continuous advancements in AI technology, our findings collectively emphasize the importance of educators remaining vigilant about how new versions may enhance or limit the usefulness of these tools in academic settings.

### 4.1 Limitations

Several limitations can be highlighted in the current study. First, this study focused on a single subject (cardiovascular pharmacology), limiting generalizability of our findings to other disciplines. While this is an ideal context given the topic’s complexity, expanding the evaluation to other healthcare disciplines could provide a broader understanding of AI’s potential and limitations in education.

Additionally, only the free versions of the AI tools were used in this study. It is possible that paid or more advanced versions could offer better performance, particularly on complex topics. However, we anticipated that the majority of students and educators predominantly rely on the free versions of these tools, as they currently appear to adequately meet users’ current needs. Therefore, we limited our research to the free AI versions.

Another limitation to consider when comparing responses across LLMs or evaluating responses generated by a single LLM is the influence of the prompt’s structure and wording. The way a prompt is crafted can significantly impact the relevance, accuracy, and utility of the AI’s response. Carefully designed prompts that align with the desired output can enhance the performance of the model and potentially lead to substantial variation in responses. This variability highlights the importance of accounting for prompt design when assessing the effectiveness and comparability of these tools.

There is also a vast and continually expanding range of generative AI applications and plugins, and this study focused on only a few selected platforms. Other models may be better suited to specific assessment types or subject areas, potentially leading to different results.

Lastly, the rate of growth and development in AI technology is staggering and rapidly evolving (16, 35). As such, the results presented may only be temporarily applicable to the current ongoing refinement of the AI platforms tested in this study.

### 4.2 Perspectives

The results of this study have important implications for the use of AI in healthcare education. While AI tools demonstrate considerable promise in assisting with basic factual learning, their limitations become more pronounced, at least partially, as the complexity of the material increases. Given the essential role that cardiovascular pharmacology plays in healthcare education and clinical practice, ensuring that students and knowledge seekers are equipped with accurate, comprehensive information is crucial. Although AI tools can supplement learning by providing quick, reliable answers to a range of questions, educators and students should exercise caution when relying on AI for more complex tasks. As demonstrated in this study, despite the overall powerful performance of ChatGPT and Copilot, they appear to have limitations with accurately providing true-or-false outcomes for questions with definitive answers when the prompt difficulty increases. As such, AI-generated information must be critically evaluated, particularly when used in contexts requiring clinical decision-making, where incorrect or incomplete information could have significant consequences. Educators should therefore use AI as a supplement rather than a replacement for traditional learning approaches, especially when students are dealing with advanced or clinical-level material.

This study highlights some avenues for future research. As AI continues to evolve, ongoing evaluation of these tools across various disciplines and how they might best be integrated into educational environments will be crucial. The significant difference in performance between AI models indicates that not all tools are equally suited for educational purposes, particularly in specialized fields such as pharmacology. Future studies could investigate how additional training or fine-tuning of AI models in specific domains could improve their performance on more advanced material.

## Data Availability

The raw data supporting the conclusions of this article will be made available by the authors, without undue reservation.
