# Supplementary material for: Artificial intelligence in healthcare education: evaluating the accuracy of ChatGPT, Copilot, and Google Gemini in cardiovascular pharmacology
Source: Front Med (Lausanne). 2025 Feb 19;12:1495378. doi: 10.3389/fmed.2025.1495378 (PMC11879995; doi:10.3389/fmed.2025.1495378)
Supplement: Supplementary file 1 [file Supplementary_file_1.docx]

**Online Supplement**

**Artificial intelligence in healthcare education: Evaluating the accuracy of ChatGPT, Copilot, and Google Gemini in cardiovascular pharmacology**

Ibrahim M. Salman^1*^, Omar Z. Ameer^1^, Mohammad A. Khanfar^1^ and Yee-Hsee Hsieh^2^

*^1^Department of Pharmaceutical Sciences, College of Pharmacy, Alfaisal University, Riyadh, Saudi Arabia*

*^2^Division of Pulmonary, Critical Care, and Sleep Medicine, School of Medicine, Case Western Reserve University, Cleveland, Ohio, United States*

***Author for correspondence**

Ibrahim M. Salman, PhD

Department of Pharmaceutical Sciences, College of Pharmacy, Alfaisal University, 11533 Riyadh, Saudi Arabia

Email: [isalman@alfaisal.edu](mailto:isalman@alfaisal.edu)

Phone: +966 11 215 8819

**Examples of multiple-choice questions (MCQs)**

Prompt: The following MCQ-type questions are designed to assess the accuracy of AI-generated answers. The goal is to ensure that AI can effectively assist healthcare students, including pharmacy, medical, nursing and allied health students, in their studies without making errors in coursework-related questions. Select the best answer for each question:

**Easy**

Which antihypertensive medication can cause the rare side effect of angioedema?

A. Nifedipine

B. Fosinopril

C. Prazosin

D. Propranolol

E. Clonidine

**Intermediate**

Outpatient prophylaxis of a patient with ventricular tachycardia is best accomplished with the administration of which of the following?

A. Adenosine

B. Diltiazem

C. Esmolol

D. Lidocaine

E. Mexiletine

**Advanced**

For the following MCQs, use one of the options below to select the right answer:

A) Only 1 and 2 are correct.

B) Only 3 is correct.

C) Only 4 and 5 are correct.

D) All statements are correct.

E) None of the statements are correct.

An elderly patient with a history of heart disease has difficulty breathing and is diagnosed with acute pulmonary edema. Which treatment is indicated to provide quick relief?

1. Acetazolamide

2. Chlorthalidone

3. Spironolactone

4. Digoxin

5. Nadolol

**Examples of short-answer questions (SAQs)**

Prompt: The following SAQ-type questions are designed to assess the accuracy of AI-generated answers. The aim is to ensure that AI can assist healthcare students including pharmacy, nursing, medical, and allied health students in studying cardiovascular pharmacology without making errors on course-related questions. Respond as a pharmacology student taking an exam, providing concise answers to the following questions. Where applicable, include a mechanistic explanation.

**Easy**

Why is propranolol contraindicated in asthma?

**Intermediate**

What is the mechanism of amlodipine-induced peripheral edema and how can it be minimized?

**Advanced**

A 65-year-old woman presented at the emergency department with atrial fibrillation (Heart rate = 180 bpm). Her past medical history includes hypertension, hyperlipidemia, hypothyroidism and mild asthma, for which she has been managed by candesartan (16 mg tablet daily), rosuvastatin (10 mg tablet daily), thyroxine (0.5 μg tablet daily) and salbutamol inhaler (as required), respectively. Her doctor ordered an intravenous loading dose of Drug X (5 mg/kg) given over 1 hour and discharged her the next day on an oral formula of Drug X (400 mg tablets BID) and aspirin (81 mg tablets daily). Six months later the woman presented with increased thyroid stimulating hormone levels and impaired asthma control. What antiarrhythmic drug was Drug X and why did it cause these symptoms? During the same doctor visit, the woman also presented with upper respiratory tract infection and her doctor decided to place her on a 5-day course of clarithromycin (500 mg tablets TID). Explain why clarithromycin would be an unsuitable pharmacological choice in this patient.

**Table (S1)**: MCQ test scores from explored AI platforms, expressed as frequencies and percentages, showing both overall performance and performance across each difficulty level.

| **AI tool** | **Answer** | **Easy**  **(*n*=15)** | **Intermediate**  **(*n*=15)** | **Advanced**  **(*n*=15)** | **Total**  **(*n*=45)** |
| --- | --- | --- | --- | --- | --- |
| ChatGPT | Correct, *n* (%) | 15 (100) | 14 (93) | 11 (73) | 40 (89) |
|  | Incorrect, *n* (%) | 0 (0) | 1 (7) | 4 (27) | 5 (11) |
| Copilot | Correct, *n* (%) | 14 (93) | 14 (93) | 8 (53) | 36 (80) |
|  | Incorrect, *n* (%) | 1 (7) | 1 (7) | 7 (47) | 9 (20) |
| Gemini | Correct, *n* (%) | 13 (87) | 14 (93) | 3 (20) | 30 (67) |
|  | Incorrect, *n* (%) | 2 (13) | 1 (7) | 12 (80) | 15 (33) |

**Table (S2)**: SAQ test scores from explored AI platforms, expressed as a score out of 5 points and as percentages, showing both overall performance and performance across each difficulty level.

| **AI tool** | **Score** | **Easy**  **(*n*=10)** | **Intermediate**  **(*n*=10)** | **Advanced**  **(*n*=10)** | **Total**  **(*n*=30)** |
| --- | --- | --- | --- | --- | --- |
| ChatGPT | Out of 5 | 4.7 ± 0.4 | 4.7 ± 0.2 | 4.7 ± 0.3 | 4.7 ± 0.3 |
|  | Percent (%) | 95 ± 8 | 95 ± 4 | 94 ± 6 | 95 ± 6 |
| Copilot | Out of 5 | 4.6 ± 0.3 | 4.6 ± 0.3 | 4.3 ± 0.6 | 4.5 ± 0.4 |
|  | Percent (%) | 92 ± 6 | 93 ± 6 | 85 ± 11 | 90 ± 9 |
| Gemini | Out of 5 | 3.5 ± 1.4 | 3.7 ± 0.6 | 2.8 ± 0.8 | 3.3 ± 1.0 |
|  | Percent (%) | 71 ± 28 | 74 ± 12 | 55 ± 15 | 67 ± 21 |

Data are expressed as mean ± SD
